# A retrospective study of internal small bowel herniation following pelvic lymphadenectomy for gynecologic carcinomas

**DOI:** 10.1038/s41598-021-81160-4

**Published:** 2021-01-14

**Authors:** Yuji Tanaka, Yusuke Shimizu, Ai Ikki, Kota Okamoto, Atsushi Fusegi, Makoto Nakabayashi, Makiko Omi, Tomoko Kurita, Terumi Tanigawa, Yoichi Aoki, Sachiho Netsu, Mayu Yunokawa, Hidetaka Nomura, Maki Matoda, Sanshiro Okamoto, Kohei Omatsu, Hiroyuki Kanao

**Affiliations:** Department of Gynecologic Oncology, Cancer Institute Hospital of JFCR, Koutouku, 3-8-31, Ariake, Koto, Tokyo, 135-8550 Japan

**Keywords:** Cancer, Diseases, Gastroenterology, Oncology, Risk factors, Signs and symptoms

## Abstract

After pelvic lymphadenectomy (PLA), pelvic vessels, nerve, and ureter are skeletonized. Internal hernias beneath the skeletonized pelvic structure following pelvic lymphadenectomy (IBSPP) are a rare complication following PLA. To the best of our knowledge, only 12 IBSPP cases have been reported and clinical details on such hernias remain unknown. The aim of the study was to investigate the incident and etiology of IBSPP. 1313 patients who underwent open or laparoscopic pelvic lymphadenectomy were identified from our database. A retrospective review was performed. Mean follow-up period was 33.9 months. A total of 12 patients had IBSPP. Multivariate analysis of laparoscopic surgeries group as compared to open surgeries group, para-aortic lymphadenectomy rate, number of dissected lymph nodes by PLA, antiadhesive material use rate, and blood loss were lower in laparoscopic surgeries group: odd ratio (OR) = 0.13 [95% confidence interval (CI) 0.08–0.19], and OR = 0.70 [95% CI 0.50–0.99], OR = 0.17 [95% CI 0.10–0.28], OR = 0.93 [95% CI 0.92–0.94]. However, no significant difference was observed in the incidence of IBSPP between laparoscopic surgery (1.0%) and open surgery (0.8%). All IBSPP occurred in the right pelvic space. These findings may contribute to the development of prevention methods for this disease.

## Introduction

In gynecological surgery, pelvic lymphadenectomy (PLA) is a typical surgical procedure that is employed for the standard treatment of ovarian, endometrial, and cervical cancer^1–3^. Small bowel obstruction is a relatively common complication following PLA; however, most of them are adhesive obstructions, and small bowel obstruction due to internal hernia is extremely rare.

In general, internal hernia is responsible for approximately 6% of small bowel obstructions, with a total incidence of 0.2–0.9%^4^. An internal hernia is defined as a protrusion of a viscus through a normal or abnormal orifice within the abdominal cavity^5,6^. Such an orifice may form as a consequence of abdominal surgery. Most well investigated iatrogenic internal hernia is “Petersen’s space hernia,” and is caused by the herniation of intestinal loops through the defect between the small bowel limbs, transverse mesocolon, and retroperitoneum after any type of gastrojejunostomy. The incidence of Petersen’s space hernia has been reported as about 2% and increases with laparoscopic surgery^7^ Closure of all mesenteric defects with permanent running sutures is a common procedure for the prevention of Petersen’s space hernia^7^.

After pelvic lymphadenectomy, pelvic vessels, nerve, and ureter are skeletonized. Internal small bowel herniation caused by the skeletonized pelvic structure as a hernia orifice is a rare complication following pelvic lymphadenectomy. To the best of our knowledge, only 12 cases of such internal hernia after PLA have been reported^4,8–17^. The primary diseases in these cases include gynecological cancer (four cases)^10–12,14^, testicular tumor (one case)^9^, prostate cancer (three cases)^4,15,16^, bladder cancer (one case)^13^, and rectal cancer (three cases)^8,17^. The common sites, frequency, risk factors, etiologies, and preventive measures for such internal hernia are unknown. For these reasons, we sought to investigate the incidence and risk factors for internal hernia. This is the first report of a retrospective study of internal hernia beneath the skeletonized pelvic structure following PLA (IBSPP).

## Methods

### Patient population

We retrospectively searched our patient database for the records of 1313 women with primary gynecological cancer who underwent pelvic lymphadenectomy and hysterectomy or trachelectomy and/or para-aortic lymphadenectomy (PALA) and/or omentectomy at the Cancer Institute Hospital of JFCR, from January 1, 2015, to February 29, 2020, and retrospective review was performed. In these 1313 patients, none received sentinel node procedures. The study was approved by the the Cancer Institute Hospital of JFCR Review Board and was performed in accordance with relevant guidelines and regulations of the institutional review board. Participants provided signed informed consent.

### Surgical technique of PLA

**Figure 1:**
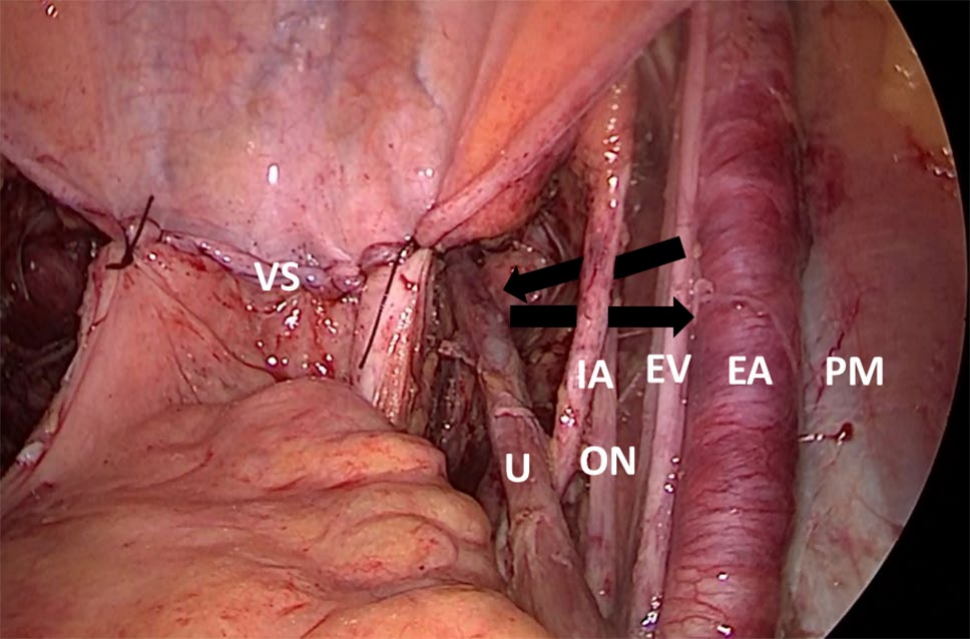
Operative findings of pelvis at the end of surgery. Internal iliac, external iliac, obturator, common iliac vessels, and obturator nerves and ureters were completely skeletonized. The retroperitoneum was left open. Arrows indicate a representative IBSPP hernial orifice. PM, Psoas muscle; EA, External iliac artery; EV, External iliac vein; IA, Internal iliac artery; ON, Obturator nerve; U, Ureter; VS, vaginal stump covered by peritoneum. This figure was drawn by corresponding author using Microsoft PowerPoint 2013 (https://www.microsoft.com/ja-jp/microsoft-365/previous-versions/microsoft-powerpoint-2013).

In all cases of pelvic lymphadenectomy, tissues along the internal iliac, external iliac, obturator, common iliac vessels, and obturator nerve were completely removed so that these vessels and nerve and ureter were completely skeletonized (Fig. 1). Retroperitoneal suction drainage was performed in all the patients. The choice of affixing or sparing antiadhesive material onto a skeletonized pelvic structure after PLA was based on each surgeon’s preference. The retroperitoneum was left open at the end of surgery in all patients.

### Follow-up

As a follow-up of cancer, each case is visited to hospital every few months. If there are symptoms such as abdominal pain or vomiting, or suspicious of recurrence of malignant tumor, CT scan was done. Even if there are no such symptoms, CT scan was done every six months. The follow-up period is determined according to the risk of recurrence in each case.

### Definition of IBSPP

IBSPP was defined as an internal hernia suspected preoperatively based on imaging and clinical symptoms and a surgeon-diagnosed internal hernia at the time of surgery that was caused by skeletonized pelvic vessels, nerve, or ureter following PLA.

### Statistical analyses

Comparison between groups was performed using the chi-squared test. Means were compared using the T-test or Mann–Whitney U test. All continuous variables were expressed as mean ± standard deviation. Comparison between open surgery and laparoscopic surgery are studied using multiple logistic regression analysis. A *p*-value < 0.05 was considered to indicate statistical significance. Results were analyzed using the Prism version 6.0 software (GraphPad, USA).

### Ethics approval and consent to participate and consent for publication

All subjects gave their informed consent for inclusion before they participated in the study. The study was conducted in accordance with the Declaration of Helsinki and the protocol was approved by the Ethics Committee of Cancer Institute Hospital of JFCR (Approval No. 2020-1033).

## Results

**Figure 2:**
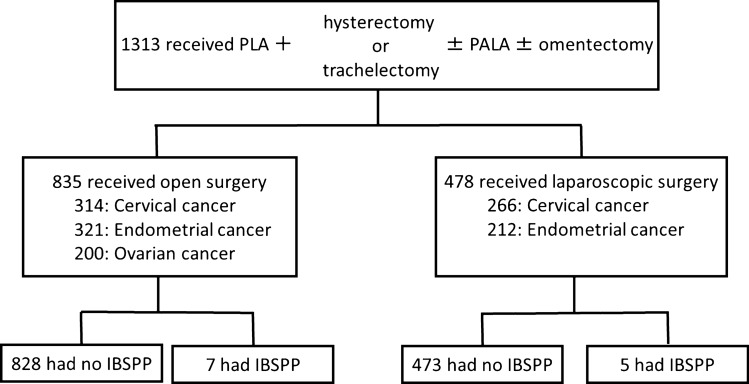
Flow chart of all patients’ contents. PLA, pelvic lymphadenectomy; PALA, para-aortic lymphadenectomy.

Among the 1313 women in this study, 12 (0.91%) had IBSPP (Fig. 2). Clinical and surgical factors were compared between subjects with IBSPP and those without IBSPP (Table 1). No significant differences were observed in age, primary disease, PALA rate, number of lymph nodes dissected by PLA, blood loss, operation time, or laparoscopic surgery rate between these groups.

**Table 1:** Clinical and surgical factors related to IBSPP.

	Without IBSPP (n = 1301)	With IBSPP (n = 12)	*p*-value
Age (years)	51.0 ± 11.5	49.2 ± 11.8	0.61
PALA rate^a^ (%)	61.9%	50%	0.58
No. of dissected lymph nodes by PLA	35.8 ± 10.2	32.7 ± 10.8	0.32
Antiadhesive material use rate^b^ (%)	91.6%	91.6%	0.99
Blood loss (ml)	488 ± 491	422 ± 434	0.60
Operation time (min)	389 ± 107	411 ± 121	0.70
Laparoscopic surgery rate^c^ (%)	36.3%	41.6%	0.70
Follow-up period (months)^d^	33.6 ± 18.0	33.9 ± 17.7	0.70

^a^Ratio of the number of PALA cases divided by the total number of cases.

^b^Ratio of the number of cases which antiadhesive material was affixed or sprayed beneath a skeletonized pelvic structure after PLA cases divided by the total number of cases.

^c^Ratio of the number of laparoscopic surgery cases divided by the total number of cases.

^d^The period from primary surgery to last visit of institution.

Clinical and surgical factors and IBSPP incidence were compared between patients who underwent open surgery and those who underwent laparoscopic surgery (Tables 2, 3). Multivariate analysis of laparoscopic surgeries group as compared to open surgeries group, para-aortic lymphadenectomy rate, number of dissected lymph nodes by PLA, antiadhesive material use rate, and blood loss were lower in laparoscopic surgeries group: odd ratio (OR) = 0.13 [95% confidence interval (CI) 0.08–0.19], and OR = 0.70 [95% CI 0.50–0.99], OR = 0.17 [95% CI 0.10–0.28], OR = 0.93 [95% CI 0.92–0.94] There was no significant difference in the incidence of IBSPP between open and laparoscopic cases (Table 2).

**Table 2:** Clinical and surgical factors and incidence of IBSPP in each surgical procedure.

	Open surgery (n = 835)	Laparoscopic surgery (n = 478)	*p*-value
Age (years)	52.3 ± 11.2	48.6 ± 11.8	< 0.001
PALA rate^a^ (%)	60.1% (502)	11.2% (54)	< 0.001
No. of dissected lymph nodes by PLA	36.4 ± 10.7	34.5 ± 9.3	0.0018
Antiadhesive material use rate^b^ (%)	96%	83%	< 0.001
Blood loss (ml)	679 ± 511	151 ± 165	< 0.001
Operation time (min)	409 ± 109	354 ± 93	< 0.001
Incidence of IBSPP (%)	0.8%	1.0%	0.62
Follow-up period (months)^c^	33.4 ± 18.2	34.7 ± 16.9	0.20

^a^Ratio of the number of PALA cases divided by the total number of cases.

^b^Ratio of the number of cases which antiadhesive material was affixed or sprayed beneath a skeletonized pelvic structure after PLA cases divided by the total number of cases.

^c^The period from primary surgery to last visit of institution.

**Table 3:** Multivariate analysis of laparoscopic surgeries group compared with open surgeries group.

	OR^a^	95% CI^b^	*p*-value
Age^c^	0.92	0.79–1.07	0.27
PALA rate	0.13	0.08–0.19	< 0.001
No. of dissected lymph nodes by PLA^d^	0.70	0.50–0.99	0.046
Antiadhesive material use rate	0.17	0.10–0.28	< 0.001
Blood loss^e^	0.93	0.92–0.94	< 0.001
Operation time (hours)	1.21	1.07–1.37	0.0014

^a^Odd ratio.

^b^Confidence interval.

^c^1 unit = 10 years.

^d^No. of dissected lymph nodes by PLA ≧ 35.

^e^1 unit = 10 mL.

Patients’ initial surgical characteristics are presented in Table 4. There were seven laparotomy cases and five laparoscopic surgery cases. PALA was performed in half of all cases. No significant difference was observed in the number of lymph nodes removed between the right and left PLAs (*p* > 0.05). In most cases (91.6%), antiadhesive material was used on skeletonized pelvic structures following PLA. In all cases, skeletonized pelvic structures were not closed after PLA. In eight cases (66.7%), adjuvant chemotherapy or radiotherapy was administered between initial operations and IBSPP operations.

**Table 4:** Initial surgical characteristics in cases of postoperative IBSPP.

No	Age (y)	Diagnosis	Stage	Operative approach	Operative method	PALA^a^	Rt PLA^b^	Lt PLA^c^	PLA^d^	Operation time (min)	Blood loss	Antiadhesive material/peritoneal suture^e^	Adj^f^
1	54	Cx Ca	IB1	Open	RH + BSO	0	12	12	24	305	330	Yes/No	Chemo
2	58	Em Ca	IB	TL	mRH + BSO	25	11	16	27	368	25	Yes/No	Chemo
3	49	Cx Ca	IIB	Open	RH + BSO	36	25	29	54	540	470	Yes/No	Radio
4	34	Cx Ca	IB1	TL	RH + BSO	0	12	13	25	358	50	Yes/No	Chemo
5	38	Cx Ca	IB1	TL	RH + BS	0	23	21	44	300	200	Yes/No	None
6	55	Ova Ca	IIIA2	Open	SH + BSO + OM	19	10	12	22	387	200	Yes/No	Chemo
7	46	Cx Ca	IB1	TL	RH + BSO	0	14	17	31	541	200	No/No	Chemo
8	69	Em Ca	IB	Open	mRH + BSO	13	10	12	22	268	750	Yes/No	Chemo
9	36	Cx Ca	IB1	Open	RH + BSO	0	20	22	42	690	480	Yes/No	Chemo
10	40	Cx Ca	IB1	LA	RT	0	20	22	42	375	65	Yes/No	None
11	45	Em Ca	IB	Open	mRH + BSO + OM	15	15	21	36	418	750	Yes/No	None
12	68	Em Ca	IA	Open	mRH + BSO	16	14	12	26	382	1550	Yes/No	None

Cx Ca, cervical cancer; Em Ca, endometrial cancer; Ova Ca, ovarian cancer; TL, total laparoscopic surgery; LA, laparoscopic-assisted surgery; RH, radical hysterectomy; BSO, bilateral salpingo-oophorectomy; SH, simple hysterectomy; mRH, modified radical hysterectomy; RT, radical trachelectomy; OM, omentectomy;

^a^Number of lymph nodes removed by para-aortic lymph node dissection.

^b^Number of lymph nodes removed by right pelvic lymph node dissection.

^c^Number of lymph nodes removed by left pelvic lymph node dissection.

^d^Total number of lymph nodes removed by pelvic lymph node dissection.

^e^Is antiadhesive material affixed or sprayed onto a skeletonized pelvic structure after PLA?/Is the skeletonized pelvic structure after PLA closed using some techniques, such as peritoneal suture?

^f^Adj, Adjuvant therapy after initial operation and before internal herniation.

**Figure 3:**
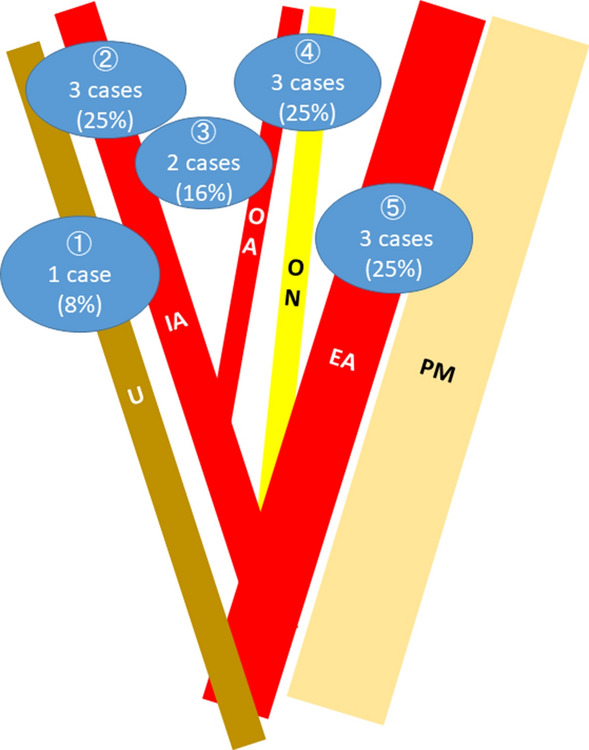
Schema of IBSPP hernial orifices. Hernial orifices of IBSPP are presented. All cases were in the right lymph node dissection space. In one case, the detailed onset site was unclear. PM, Psoas muscle; EA, External iliac artery; IA, Internal iliac artery; OA, Obturator artery; ON, Obturator nerve; U, Ureter. (1) Caused by the right ureter. (2) Caused by the right Internal iliac artery. (3) Caused by the right triangle space of the internal iliac artery and obturator artery. (4) Caused by the right obturator artery. (5) Caused by the right external iliac artery. This figure was drawn by corresponding author using Microsoft PowerPoint 2013 (https://www.microsoft.com/ja-jp/microsoft-365/previous-versions/microsoft-powerpoint-2013).

Surgical findings at the time of IBSPP operation are presented in Table 5. The median interval time between operation and onset of obstruction was 13.5 months. All IBSPP occurred in the right pelvic space and did not occur on the left side. Hernial orifices are presented in Fig. 3. Four cases (33.3%) were relative to the obturator artery. Excluding unclear cases, the small bowel herniation point was within 20 cm of the ileocecal valve in four cases (57.1%). There was no recurrence of internal hernia in any of the cases. Four cases (33.3%) received laparoscopic surgery; however, one case required conversion from laparoscopic surgery to laparotomy. Seven cases (58.3%) required bowel resection. Excluding unclear cases, resection of the obturator or umbilical artery was performed in three cases (30%), four cases (40%) were closed by mobilizing the caecum or sigmoid colon, and two cases (20%) were unrepaired.

**Table 5:** The surgical findings at IBSPP repair operation.

No	Interval months^a^	Hernial orifice		Herniation point of small bowel^b^	Operative approach	Bowel resection, resection length	Orifice repair method	POS (day)^c^	postop complication^d^
1	36	Right	Obturator artery	3 m from Treitz's ligament	Open	No	Resection of the obturator artery	24	Ileus (paralytic)
2	15	Right	Umbilical artery	Unclear	Open	Ileum, 25 cm	Unclear	14	No
3	2	Right	External iliac artery	20 cm from the ileocecal	Open	Ileum, 20 cm	Unrepaired	18	No
4	44	Right	Between Umbilical artery and obturator artery	4 cm from the ileocecal	Open	Ileum, 50 cm	With sigmoid colon	7	No
5	7	Right	Between Umbilical artery and obturator nerve	5 cm from the ileocecal	TL	No	Resection of the umbilical artery	7	No
6	29	Right	Unclear	5 cm from the ileocecal	Open	Ileocecum	Unclear	48	Intra-abdominal abscess
7	18	Right	Umbilical artery	50 cm from the ileocecal	Open	Ileum	Unrepaired	14	No
8	20	Right	Ureter	Unclear	Open	No	With sigmoid colon	12	No
9	12	Right	Obturator artery	30 cm from the ileocecal	Open	Ileum, 11 cm	Unrepaired	8	No
10	9	Right	Obturator artery	Unclear	Conversion^c^	Ileum, 50 cm	Resection of the obturator artery	10	No
11	1	Right	External iliac artery	Unclear	TL	No	With sigmoid colon	8	No
12	1	Right	Umbilical artery	Unclear	TL	No	With ileum	10	No

TL, total laparoscopic surgery.

^a^Interval months between operation and onset of obstruction.

^b^Position of the incarcerated small bowel.

^c^Length of postoperative hospital stay.

^d^The Clavien-Dindo scale was used to evaluate postoperative complications, and IIIa or higher was defined as major complications.

## Discussion

This is the first retrospective study on internal hernia beneath skeletonized pelvic structures following pelvic lymph node dissection. We discuss the incidence, risk factors, etiology, repair method, and prevention methods of IBSPP.

We describe about risk factors for IBSPP. Previous studies have suggested the possibility of increased incidence of IBSPP with laparoscopic surgery^8,14^. This is because low adhesions are a risk factor for internal hernia. However, in this study, no significant difference was observed in the incidence of IBSPP between laparoscopic and open surgeries. In the laparoscopic surgeries group, there was lower operative stress to be expressed due to lower blood loss, PALA rate, number of dissected lymph nodes by PLA, (this mean lower internal hernia risk due to low adhesion), and lower rate of antiadhesion material use (lower internal hernia risk due to high adhesion). As a result, the risk of IBSPP might be offset.

We describe about the etiology of IBSPP. The most significant finding in this study is that all cases occurred on the right side. This finding has implication on the investigation of internal hernia prevention measures. We have inferred that there are two causes for this asymmetry. First, peristalsis of the small intestine was from the ligament of Treitz (abdominal midline) to the ileocecum, which is on the right side of the abdomen near the right skeletonized pelvic structure. Therefore, IBSPP tends to occur on the right side. Second, the left skeletonized pelvic structure was covered by the sigmoid colon following PLA. Therefore, IBSPP tends not to occur on the left side. The strangulated small intestine was often close to the ileocecal region. This was probably because the dissection site of the right lymph node was close to this region.

We describe about IBSPP repair surgery. Several techniques for closing orifices following PLA can be discussed. Closing the orifice with peritoneum flap and/or sigmoid colon and/or ileocecum^10,13^ has been thought to be the most convenient method. However, other techniques are sometimes required due to limited peritoneum around the skeletonized structure. Closing the orifice using a free peritoneal graft^9^ or by gluing a collagen patch^12^ or mesh^11^ with running absorbable surgical sutures may be secondary options. If the causal structure is unimportant, such as the obturator artery, resection of the causal structure^14^ is an option.

We describe about preventive methods for internal hernia. Peritoneal closure of the pelvic lymph node dissection space might prevent IBPSS. Because bilateral peritoneal closure may increase the frequency of lymphoma cysts^18–20^ and IBSPP tends to occur on the right side, right-side closure only may be sufficient. Right-side space closure by mobilizing the caecum might be effective and low invasive method. More sentinel node procedures might be most direct prevention method of IBSPP.

This study has some limitations. Larger sample sizes may be needed to delineate the risk factors for IBSPP. Larger sample sizes may reveal the statistical risk factors for IBSPP; however, event size is limited by low incidence. It was not possible to investigate what preventive measures would reduce the incidence of IBSPP; however, our findings of IBSPP tending to occur on the right side may help develop beneficial precautions. A prospective study of whether various preventive measures can prevent internal hernia is desired.

In conclusion, IBSPP tends to occur on the right side. The incidence of IBSPP is not different in both open and laparoscopic surgery significantly. These findings may contribute to the development of prevention methods for this disease.

## Data Availability

The datasets analyzed during the current study are available from the corresponding author on reasonable request.

## Code availability

The software application/code used during the current study is available from the corresponding author on reasonable request. All the experiment protocol for involving human data was in accordance to guidelines of institutional Declaration of Helsinki.
